# Etiological, sociodemographic and clinical characteristics of sexually transmitted infections and *M. genitalium* resistance in Shenzhen: a multicenter cross-sectional study in China

**DOI:** 10.3389/fcimb.2024.1407124

**Published:** 2024-07-25

**Authors:** Feng Wang, Chi Zhang, Leshan Xiu, Yamei Li, Yaling Zeng, Yizhun Li, Yumao Cai, Junping Peng

**Affiliations:** ^1^ Shenzhen Institute of Dermatology, Shenzhen Center for Chronic Disease Control, Shenzhen, China; ^2^ NHC Key Laboratory of Systems Biology of Pathogens, National Institute of Pathogen Biology, Chinese Academy of Medical Sciences & Peking Union Medical College, Beijing, China; ^3^ National Institute of Pathogen Biology and Key Laboratory of Respiratory Disease Pathogenomics, Chinese Academy of Medical Sciences & Peking Union Medical College, Beijing, China; ^4^ Key Laboratory of Pathogen Infection Prevention and Control (Ministry of Education), State Key Laboratory of Respiratory Health and Multimorbidity, National Institute of Pathogen Biology, Chinese Academy of Medical Sciences& Peking Union Medical College, Beijing, China

**Keywords:** sexually transmitted infections, sociodemographic characteristics, urogenital symptoms, *Mycoplasma genitalium*, antimicrobial resistance

## Abstract

**Introduction:**

This study aims to determine the etiological, sociodemographic, and clinical characteristics of STIs, and the level of resistance in *M. genitalium* in Shenzhen, a representative first-tier city of southern China.

**Methods:**

A multicenter cross-sectional study was conducted and 7886 sexually active participants attending STI-related departments were involved from 22 hospitals. Nine STI-related organisms including *N. gonorrhoeae*, *C. trachomatis*, *T. vaginalis*, *M. genitalium*, HSV-1, HSV-2, *M. hominis*, *U. parvum*, and *U. urealyticum* were screened.

**Results:**

Being single or divorced was associated with increased detection of *N. gonorrhoeae*, C*. trachomatis*, *M. genitalium*, HSV-1, HSV-2 and *M. hominis*. Lower education level was associated with increased detection of *C. trachomatis*, HSV-2 and *M. hominis*. No insurance coverage was an independent risk factor for *T. vaginalis*, *M. hominis* and *U. parvum* positivity. Three resistance-determining regions related to macrolide and fluoroquinolone were sequenced in 154 *M. genitalium* positive samples, among which 90.3% harbored mutations related to macrolide or fluroquinolone resistance and 67.5% were multidrug-resistant *M. genitalium*. A2072G in 23S rRNA and Ser83Ile in *parC* were the most common mutations. *M. hominis* was associated with manifestations of bacterial vaginosis in female and epididymitis in male.

**Conclusions:**

Single or divorced individuals, those with lower education level and individuals without insurance are higher-risk key populations for STIs. The prevalence of antimicrobial-resistant *M. genitalium* in Shenzhen is high. Detection of *M. hominis* increased significantly with lower education level and no health insurance coverage, and it is associated with bacterial vaginosis or epididymitis, indicating that *M. hominis* deserves further attention.

## 1 Introduction

The high burden of sexually transmitted infections (STIs) remains a current public health issue worldwide. In 2019, there were 769.85 million incident cases of STIs globally (Zheng et al., 2022), although less likely to be fatal, they seriously affect the quality of life and lead to considerable neonatal deaths and infertility. Besides gonorrhea and chlamydia, infections of antimicrobial-resistant *Mycoplasma genitalium* and herpes simplex virus (HSV) are also emerging global health problem. *Mycoplasma hominis*, *Ureaplasma parvum* and *Ureaplasma urealyticum* are microorganisms frequently found in human urogenital tract and their roles in causing specific symptoms of STIs remains controversial (Horner et al., 2018).

The prevalence of STI-related organisms varies globally, demonstrating the regional differences in socioeconomics, demographics, sexual orientation and cultural factors (World Health Organization, 2016; Van Gerwen et al., 2022). To date, studies in China mainly described the prevalence of common STI pathogens with limited emphasis on their correlation with sociodemographic characteristics and clinical presentations (Yin et al., 2018; Li et al., 2020; Han et al., 2021). The absence of these data undermines the formulation of region-specific, evidence-based interventions and policies.

In this study, a large-scale screening of STI-related organisms was performed in Shenzhen, a representative first-tier city of southern China. The participants included healthy and symptomatic individuals of both genders with varied symptoms of STI. Nine STI-related organisms (*N. gonorrhoeae*, *C. trachomatis*, *Trichomonas vaginalis*, *M. genitalium*, HSV-1, HSV-2, *M. hominis*, *U. parvum*, *U. urealyticum*) were detected using a multiplex PCR coupled with mass spectrum method, STI-MS (sexually transmitted infection-mass spectrometry) (Xiu et al., 2019). *M. genitalium*-positive samples underwent screening to determine the presence of mutations associated with macrolide and fluoroquinolone resistance. The aim of the study was to determine the sociodemographic and clinical features of higher-risk key populations with different STIs, and to describe the level of resistance in *M. genitalium*. The findings can contribute to the formulation of screening strategies based on key populations and specific symptoms.

## 2 Materials and methods

### 2.1 Study design and population

**Figure 1:**
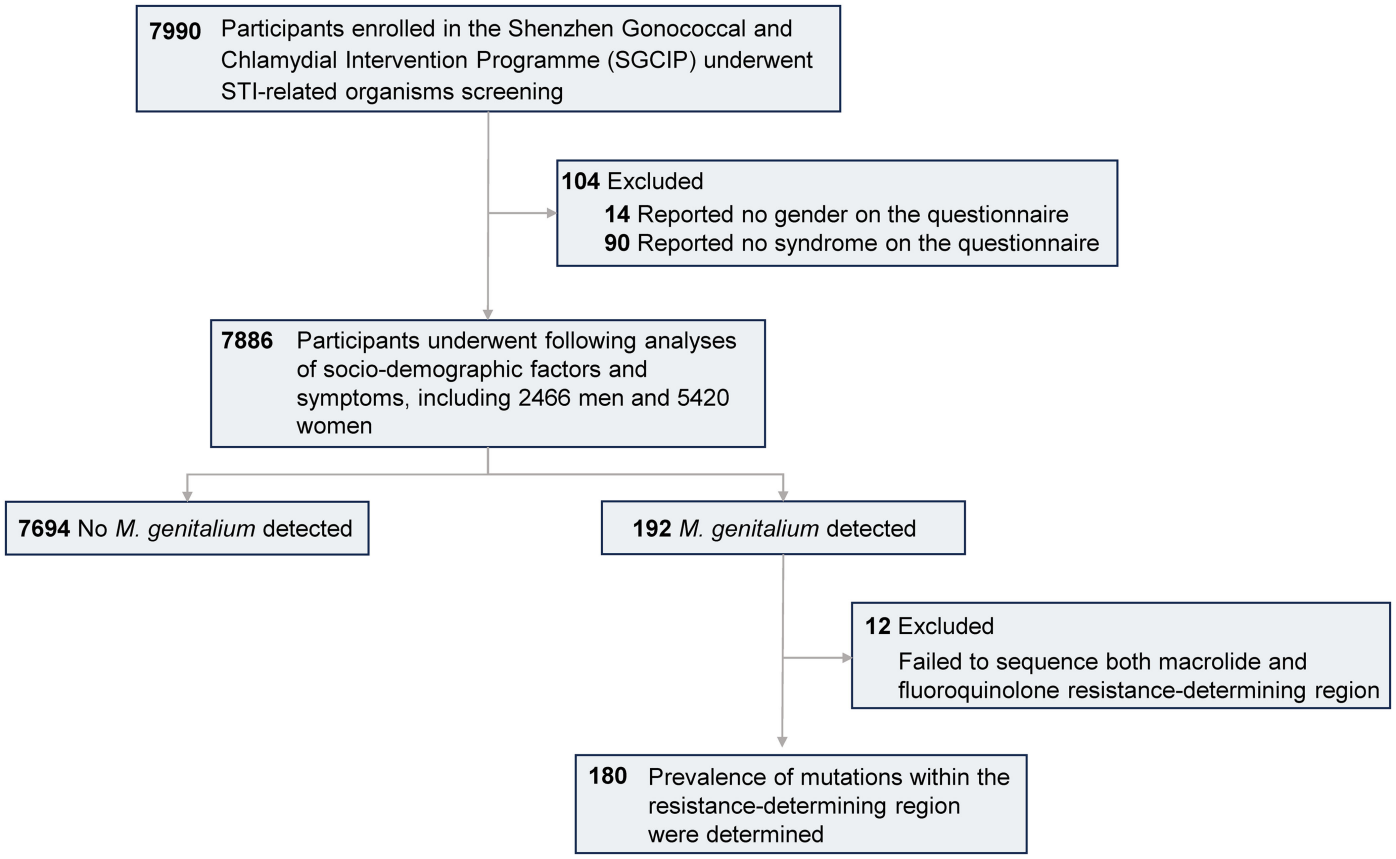
Flow diagram of study population.

This cross-sectional study was conducted among subjects enrolled in the Shenzhen Gonococcal and Chlamydial Intervention Programme (SGCIP). Twenty-two hospitals situated across six districts in Shenzhen were included in the programme (**Supplementary Table 1**). Individuals attending STI-related departments including dermatology, urology, gynecology and obstetrics in 2018 were evaluated. The inclusion criteria were as follows: aged ≥ 16 years, sexually active, and had not used antibiotics within the past two weeks. All participants completed a questionnaire regarding their sociodemographic characteristics. Urethral swabs were collected from males and cervical swabs were collected from females. Clinicians conducted physical examinations and documented the symptoms. A total of 7990 participants enrolled in the SGCIP underwent STI-related organisms screening and 104 participants who failed to correctly fill the gender and symptoms on the questionnaire were excluded. The remaining 2466 men and 5420 women were eligible (**Figure 1**). Participants without any of the above symptoms were defined as controls.

This study followed the Strengthening the Reporting of Observational Studies in Epidemiology (STROBE) reporting guideline.

### 2.2 Sample preparation and mass spectrum test

Swabs were rotated in 500μL phosphate-buffered saline and 200μL were used for DNA extraction with MagNA Pure LC 2.0 instrument and MagNA Pure LC Total Nucleic Acid Isolation Kit (Roche Diagnostics, Mannheim, Germany). *N. gonorrhoeae*, *C. trachomatis*, *T. vaginalis*, *M. genitalium*, HSV-1, HSV-2, *M. hominis*, *U. parvum*, and *U. urealyticum* were simultaneously screened using the STI-MS method (Xiu et al., 2019).

### 2.3 Sequencing of the macrolide and fluoroquinolone resistance-determining region

Sequences of the macrolide RDR (23S rRNA) and fluoroquinolone RDR (*parC* and *gyrA* gene) were amplified using nested PCR from the *M. genitalium*-positive samples. Sanger sequencing was performed on amplicons (Tsingke Biotechnology, Beijing, China). PCR reactions were conducted with the Roche FastStart High Fidelity PCR System (Roche Diagnostics). Primers were listed in **Supplementary Table 2**.

### 2.4 Statistical analysis

The 95% confidence intervals for the detection rate of each STI-related organism were calculated using the VassarStats website (http://vassarstats.net/index.html). The chi-square tests were used to compare the difference in the detection rate of each STI-related organism in the male and female populations. Sociodemographic factors associated with the detection of STI-related organism and antimicrobial-resistant *M. genitalium* were first investigated using the univariable logistic regression analyses, and factors with a significance level of P < 0.1 were included in the multivariable model. All the nine STI-related organisms detected in the study were included in the multivariable logistic regression model to investigate their associations with the urogenital symptoms of male and female participants respectively. The chi-squared tests and logistic regression analyses were performed using SPSS 26.0

## 3 Results

### 3.1 Sociodemographic and clinical characteristics

Of the 7886 eligible participants, 5420 (68.7%) were women and the median age was 31 years (interquartile range, 27-36). The majority of participants were married (74.5%), with the most common educational attainment being college or graduate level (38.2%). Most participants had health insurance (61.4%), with 16.6% reporting a monthly income of less than US$500. 96.7% of the participants were heterosexual and 36% reported having one or more casual sexual partners in the past three months. Other sociodemographic characteristics, including residency, living time in Shenzhen, and occupation, are listed in **Supplementary Table 3**.

Among eligible participants, 1290 males and 3454 females had at least one clinical sign or symptom. For male, urethral burning or irritation was the most common symptom (25.1%), while for female, the most common symptom was abnormal vaginal discharge (53.8%). The statistics of clinical signs and symptoms were listed in **Supplementary Table 4**.

### 3.2 Detection of STI-related organisms

According to the results of STI-MS, among the 7886 eligible participants, 1095 (44.4%) males and 3572 (65.9%) females were positive for at least one microorganism. *C. trachomatis* was the most common detected STI pathogen, with the detection rate of 11.6% in male and 10.0% in female; *U. parvum* was the most frequently detected STI-related organism, with the detection rate of 17.3% in male and 51.2% in female. Among STI pathogens, the prevalence of *N. gonorrhoeae* and *C. trachomatis* of men was significantly higher than women, but for *T. vaginalis*, the prevalence of women was higher. The detection rates of *M. hominis*, *U. parvum* and *U. urealyticum* in women were significantly higher than men (**Supplementary Table 5**). The coinfection rate of six STI pathogens was 4.1% (100/2466) for male and 2.6% (139/5420) for female. Taking *M. hominis*, *U. parvum* and *U. urealyticum* into account, the coinfection rate was 14.4% (355/2466) and 23.2% (1255/5420) for male and female respectively.

### 3.3 Sociodemographic characteristics associated with detection of STI-related organisms

Univariable analyses showed that *N. gonorrhoeae* and *C. trachomatis* infections were found more frequently in males than females, while for *T. vaginalis*, *H. hominis*, *U. parvum*, and *U. urealyticum*, the situation was reversed. No health insurance coverage and marital status as single or divorced emerged as risk factors for a broader spectrum of pathogen infections. Higher level of education, income and older age were primary protective factors. Casual sex partners, mobility of population (those without residency or with short living time) were associated with increased organism positivity. Compared to unemployment, government staff and housewife/househusband were linked to decreased organism positivity (**Supplementary Tables 6-8**).

Results of multivariate analyses of sociodemographic factors associated with *N. gonorrhoeae* and *C. trachomatis* infections were shown in **Supplementary Table 9**, and the findings aligned with previous studies conducted on the same population (Ning et al., 2022; Wang et al., 2022). For the other seven STI-related organisms, *T. vaginalis*, *M. hominis*, *U. parvum* and *U. urealyticum* were found more frequently among females. Being single or divorced was an important factor related to infection, which was associated with increased detection of *M. genitalium* (aOR=1.50, 95%CI: 1.04-2.17), HSV-1 (aOR=1.53, 95%CI: 1.03-2.28), HSV-2 (aOR=1.82, 95%CI: 1.20-2.75) and *M. hominis* (aOR=1.34, 95%CI: 1.12-1.61). Elevated educational level was significantly correlated with reduced odds of HSV-2 and *M. hominis* infection. No insurance coverage was an independent risk factor for *T. vaginalis*, *M. hominis* and *U. parvum* positivity. Moreover, monthly income more than $500 was associated with decreased detection of *T. vaginalis* (aOR=0.59, 95%CI: 0.38-0.92). The prevalence of *U. parvum* was higher in participants with casual sex partner in last 3 months (**Table 1**). When other sociodemographic factors were included, the association of occupation, mobility of population and sexual orientation with infections weakened (**Supplementary Table 10**).

**Table 1:** Multivariable analysis of sociodemographic factors associated with STI-related organisms.

Factors aOR (95%CI) P value	*T. vaginalis*	*M. genitalium*	HSV-1	HSV-2	*M. hominis*	*U. parvum*	*U. urealyticum*
Female	3.08 (1.73-5.49) **<0.0001**				2.56 (2.10-3.13) **<0.0001**	5.21 (4.59-5.92) **<0.0001**	1.28 (1.09-1.50) **0.003**
Age		0.98 (0.95-1.00) 0.066				1.00 (0.99-1.01) 0.97	0.99 (0.98-1.00) **0.036**
Single or divorced		1.50 (1.04-2.17) **0.031**	1.53 (1.03-2.28) **0.036**	1.82 (1.20-2.75) **0.005**	1.34 (1.12-1.61) **0.001**		1.17 (0.98-1.40) 0.087
Education							
Primary or junior high school	1			1	1		1
Senior or vocational high school	0.81 (0.50-1.30) 0.38			1.18 (0.76-1.84) 0.46	0.76 (0.63-0.92) **0.005**		0.96 (0.81-1.15) 0.69
College or graduate	0.74 (0.43-1.26) 0.27			0.48 (0.28-0.83) **0.008**	0.66 (0.53-0.82) **<0.0001**		0.89 (0.73-1.10) 0.28
No insurance coverage	1.70 (1.14-2.54) **0.009**	0.97 (0.70-1.35) 0.87	1.21 (0.84-1.76) 0.31	1.11 (0.74-1.68) 0.61	1.20 (1.02-1.41) **0.031**	1.15 (1.03-1.28) **0.010**	1.12 (0.96-1.30) 0.15
Monthly income more than $500	0.59 (0.38-0.92) **0.020**				1.11 (0.91-1.36) 0.29		0.95 (0.79-1.14) 0.58
Having casual sex partner in last 3 months		1.32 (0.98-1.79) 0.069				1.12 (1.01-1.25) **0.033**	

P value less than 0.05 were bold.

### 3.4 Detection of macrolide and fluoroquinolone resistance-associated mutations in *M. genitalium*-positive samples

Among the 192 *M. genitalium*-positive samples, successful amplification and sequencing of the macrolide RDR were achieved in 180 (93.8%) samples (**Figure 1**), with mutations in 83.9% (151/180) of these samples. Four mutations were identified and A2072G was the most common (44.4%, 80/180). Other mutations included A2071G (n=48), A2071T (n=21) and A2075T (n=2) (**Table 2**).

**Table 2:** Prevalence of mutations within the RDR among *M. genitalium*-positive samples.

Gene and amplified regions of the RDR^a^	No. (%) of samples yielded RDR sequences	Mutations^b^	No. (%) of samples with mutations within the RDR
23S rRNA (2031-2258)	180 (93.8)		
		A2071G (2058)	48 (26.7)
		A2071T (2058)	21 (11.7)
		A2072G (2059)	80 (44.4)
		A2075T (2062)	2 (1.1)
		WT	29 (16.1)
*gyrA* (171-400)	163 (84.9)		
		Met95Ile	1 (0.6)
		Asp99Tyr	2 (1.2)
		Asp107Asn	1 (0.6)
		WT	159 (97.5)
*parC* (202-382)	160 (83.3)		
		Gly81Cys	1 (0.6)
		Ser83Ile	82 (51.3)
		Ser83Asn	14 (8.8)
		Ser83Asn & Asp87Asn	1 (0.6)
		Asp87Tyr	8 (5.0)
		Asp87Asn	12 (7.5)
		WT	42 (26.3)

a M. genitalium G37 genome (NC_000908.2) is used as the reference.

b For 23S rRNA mutations, E. coli numbering is shown in parentheses. For gyrA and parC amino acid changes, positions of the M. genitalium G37 genome (NC_000908.2) are used.

WT, wild type; RDR, resistance-determining region.

Successful amplification and sequencing of the *parC* gene were achieved in 160 (83.3%) *M. genitalium*-positive samples, with missense mutations in 73.8% (118/160) of these samples. Five missense mutations were detected and Ser83Ile was the most common, with a detection rate of 51.3% (82/160). Other missense mutations included Ser83Asn (n=14), Asp87Asn (n=12), Asp87Tyr (n=8) and Gly81Cys (n=1). One sample harbored a double *parC* gene missense mutation (Ser83Asn and Asp87Asn) (**Table 2**).

For the *gyrA* gene, amplification and sequencing were successful in 163 (84.9%) *M. genitalium*-positive samples, with missense mutations in 2.5% (4/163) of these samples, including Asp99Tyr (n=2), Met95Ile (n=1) and Asp107Asn (n=1). All the samples harboring missense mutations in the *gyrA* gene exhibited missense mutations in the *parC* gene (**Table 2**).

Successful amplification and sequencing of the three RDRs were achieved in 154 samples. Among those samples, 16.2% (25/154) were macrolide-resistant *M. genitalium* infection (with mutations solely in the macrolide RDR); 6.5% (10/154) were fluoroquinolone-resistant *M. genitalium* infection (with mutations solely in the fluoroquinolone RDR); 67.5% (104/154) were multidrug-resistant *M. genitalium* infection; 9.7% (15/154) exhibited wild-type sequences of the three RDRs.

The prevalence of macrolide or fluoroquinolone-resistant *M. genitalium* infection and associations with sociodemographic factors, co-infecting organisms, and urogenital symptoms were determined with univariate and multivariate analysis. Univariate regression analysis showed participants lacking Shenzhen residency [OR=2.75 (1.08-6.99), p=0.034] and without insurance coverage [OR=2.41 (1.14-5.10), p=0.021] exhibited a higher rate of fluoroquinolone-resistant *M. genitalium* infection, while the multivariate analysis included Shenzhen residency and insurance showed the two factors were not independent for increased fluoroquinolone-resistant *M. genitalium* prevalence (p=0.138 and 0.087 respectively). No specific co-infecting organisms and urogenital symptoms were significantly associated with macrolide or fluoroquinolone-resistant *M. genitalium* infection.

### 3.5 Urogenital symptoms associated with STI-related organisms

As accepted causes of male urethritis, *N. gonorrhoeae*, *C. trachomatis*, and *M. genitalium* were associated with urethral serous discharge, urethral purulent discharge, and urethral burning or irritation. HSV-2 infection in male was strongly associated with genital or perianal blisters (aOR=11.32, 95%CI: 3.10–41.26) and ulcers (aOR=10.41, 95%CI: 2.38–45.49), indicating in males with genital lesions, if tests for pathogens that cause such lesions, including *Treponema pallidum*, are negative, HSV should be considered the primary causative organism, necessitating antiviral treatment. Furthermore, the detection of *M. hominis*, *U. parvum*, and *U. urealyticum* was found to be associated with certain urogenital symptoms. In this study, epididymis swelling or pain was solely associated with *M. hominis* (aOR=4.02, 95%CI: 1.40–11.57). *U. urealyticum* was observed to be associated with genital or perianal blisters (aOR=2.93, 95%CI: 1.63–5.24), whereas *U. parvum* was associated with a decreased risk of urethral purulent discharge (aOR=0.5, 95%CI: 0.28–0.89) (**Table 3**).

**Table 3:** Urogenital symptoms of male associated with STI-related organisms.

Microorganism aOR (95%CI) P value	Urethral serous discharge	Urethral purulent discharge	Scrotum swelling or pain	Epididymis swelling or pain	Balanopos-thitis	Urethral burning or irritation	Genital or perianal blisters	Genital or perianal ulcers	Genital or perianal warts
*N. gonorrhoeae*	3.04 (1.88-4.91) **<0.0001**	8.72 (5.72-13.29) **<0.0001**	1.38 (0.53-3.58) 0.51	0.79 (0.18-3.50) 0.76	1.56 (0.68-3.57) 0.30	2.20 (1.55-3.12) **<0.0001**	2.31 (1.07-4.97) **0.033**	3.23 (1.44-7.25) **0.004**	1.34 (0.73-2.45) 0.34
*C. trachomatis*	3.63 (2.39-5.52) **<0.0001**	3.56 (2.22-5.69) **<0.0001**	1.03 (0.40-2.65) 0.96	2.19 (0.79-6.13) 0.13	1.48 (0.68-3.25) 0.32	2.74 (2.01-3.73) **<0.0001**	1.64 (0.76-3.52) 0.21	1.20 (0.45-3.20) 0.72	1.31 (0.75-2.28) 0.34
*T. vaginalis*	1.43 (0.27-7.57) 0.68	2.01 (0.35-11.44) 0.43	NA^a^	2.45 (0.23-26.43) 0.46	2.22 (0.26-19.21) 0.47	0.51 (0.10-2.64) 0.42	NA^a^	NA^a^	NA^a^
*M. genitalium*	2.99 (1.41-6.34) **0.004**	2.88 (1.20-6.91) **0.018**	0.79 (0.10-6.01) 0.82	NA^a^	0.57 (0.07-4.38) 0.59	1.92 (1.08-3.43) **0.027**	1.01 (0.22-4.64) .99	NA^a^	1.19 (0.44-3.22) 0.73
HSV-1	2.25 (0.74-6.80) 0.15	3.13 (0.93-10.47) 0.065	1.39 (0.18-10.93) 0.75	NA^a^	1.39 (0.18-11.04) 0.76	1.43 (0.60-3.39) 0.42	0.83 (0.13-5.32) 0.84	0.74 (0.07-7.58) 0.80	1.02 (0.27-3.83) 0.97
HSV-2	2.16 (0.56-8.29) 0.26	NA^a^	NA^a^	NA^a^	NA^a^	1.59 (0.54-4.70) 0.40	11.32 (3.10-41.26) **<0.0001**	10.41 (2.38-45.49) **0.002**	2.13 (0.53-8.50) 0.29
*M. hominis*	0.90 (0.44-1.83) 0.77	0.95 (0.42-2.17) 0.91	0.64 (0.18-2.18) 0.47	4.02 (1.40-11.57) **0.010**	1.71 (0.70-4.21) 0.24	1.52 (1.00-2.30) 0.051	1.83 (0.79-4.24) 0.16	1.04 (0.29-3.75) 0.95	1.16 (0.59-2.29) 0.67
*U. parvum*	0.75 (0.47-1.20) 0.24	0.50 (0.28-0.89) **0.018**	1.63 (0.92-2.90) 0.093	0.62 (0.24-1.64) 0.34	0.61 (0.30-1.24) 0.17	0.80 (0.61-1.06) 0.12	0.63 (0.30-1.31) 0.21	0.95 (0.44-2.05) 0.90	0.98 (0.65-1.48) 0.92
*U. urealyticum*	1.55 (0.97-2.46) 0.066	1.03 (0.58-1.83) 0.91	1.05 (0.46-2.39) 0.90	0.91 (0.32-2.60) 0.86	1.53 (0.79-2.96) 0.21	0.91 (0.65-1.26) 0.56	2.93 (1.63-5.24) **<0.0001**	1.27 (0.54-2.98) 0.59	1.11 (0.67-1.83) 0.69

a Due to the absence of any detected instance in the case group, it was not possible to calculate the corresponding odds ratio (OR) value. P value less than 0.05 were bold. NA, not available.

Females demonstrated a different pattern in symptom-organism associations compared to males. Infections of causative agents of male urethritis (*N. gonorrhoeae*, *C. trachomatis*, and *M. genitalium*) were not linked to urethritis symptoms in female, indicating most infections of theses pathogens were asymptomatic. We found that HSV-1 and *M. hominis* were more frequently detected in female participants with various symptoms. Specifically, HSV-1 was associated with abnormal vaginal discharge (aOR=2.57, 95%CI: 1.33-4.96), mucopurulent cervicitis (aOR=4.31, 95%CI: 1.29-14.44), lower abdominal pain (aOR=4.10, 95%CI: 1.63-10.35), urethral burning or irritation (aOR=5.47, 95%CI: 2.07-14.47), and genital or perianal blisters (aOR=10.68, 95%CI: 1.95-58.39); *M. hominis* was correlated with abnormal vaginal discharge (aOR=1.30, 95%CI: 1.09-1.56), vaginal itch (aOR=2.22, 95%CI: 1.24-3.97), and genital or perianal blisters (aOR=2.30, 95%CI: 1.01-5.24). *U. parvum* was considered to be a protective factor of urethral burning or irritation with 0.67-fold decreased risk (95%CI: 0.5-0.89). Detailed results of symptom-organism associations of female were shown in **Table 4**.

**Table 4:** Urogenital symptoms of female associated with STI-related organisms.

Microorganism aOR (95%CI) P value	Abnormal vaginal Discharge	Mucopuru-lent cervicitis	Lower abdominal pain	Vaginal itch	Urethral burning or irritation	Genital or perianal blisters	Genital or perianal ulcers	Genital or perianal warts
*N. gonorrhoeae*	1.20 (0.77-1.85) 0.42	1.08 (0.41-2.83) 0.88	1.20 (0.58-2.48) 0.62	0.76 (0.10-5.74) 0.79	1.04 (0.39-2.78) 0.94	2.49 (0.51-12.19) 0.26	3.08 (0.66-14.34) 0.15	1.69 (0.50-5.78) 0.40
*C. trachomatis*	1.04 (0.85-1.26) 0.73	1.18 (0.77-1.81) 0.45	1.05 (0.75-1.48) 0.78	1.06 (0.51-2.21) 0.88	1.08 (0.68-1.71) 0.75	1.13 (0.42-3.05) 0.81	1.37 (0.50-3.80) 0.54	0.91 (0.45-1.83) 0.79
*T. vaginalis*	1.22 (0.79-1.89) 0.36	2.28 (1.12-4.63) **0.022**	0.73 (0.30-1.78) 0.49	0.54 (0.07-4.12) 0.55	1.88 (0.84-4.24) 0.13	2.30 (0.49-10.85) 0.29	2.15 (0.42-11.07) 0.36	0.45 (0.06-3.43) 0.44
*M. genitalium*	1.01 (0.67-1.52) 0.96	1.03 (0.44-2.44) 0.94	1.24 (0.64-2.38) 0.52	1.82 (0.60-5.52) 0.29	1.53 (0.67-3.52) 0.31	0.75 (0.09-6.44) 0.80	NA^a^	2.69 (1.06-6.83) **0.038**
HSV-1	2.57 (1.33-4.96) **0.005**	4.31 (1.29-14.44) **0.018**	4.10 (1.63-10.35) **0.003**	4.21 (0.51-34.82) 0.18	5.47 (2.07-14.47) **0.001**	10.68 (1.95-58.39) **0.006**	1.39 (0.09-22.16) 0.81	0.75 (0.06-8.98) 0.82
HSV-2	1.42 (0.76-2.65) 0.27	0.70 (0.17-2.89) 0.63	0.80 (0.27-2.40) 0.69	NA^a^	2.11 (0.75-5.94) 0.16	1.22 (0.15-10.07) 0.85	4.91 (0.67-35.68) 0.12	2.37 (0.40-13.92) 0.34
*M. hominis*	1.30 (1.09-1.56) **0.004**	1.42 (0.97-2.07) 0.072	1.10 (0.80-1.51) 0.57	2.22 (1.24-3.97) **0.007**	0.89 (0.57-1.39) 0.61	2.30 (1.01-5.24) **0.047**	1.28 (0.50-3.31) 0.61	1.71 (0.98-2.97) 0.058
*U. parvum*	1.00 (0.89-1.12) 0.99	0.82 (0.62-1.07) 0.15	0.91 (0.74-1.12) 0.37	1.08 (0.67-1.74) 0.75	0.67 (0.51-0.89) **0.005**	0.87 (0.45-1.68) 0.68	1.59 (0.77-3.28) 0.21	0.85 (0.55-1.29) 0.44
*U. urealyticum*	1.10 (0.93-1.30) 0.28	1.40 (0.99-1.99) 0.060	1.07 (0.80-1.44) 0.65	1.22 (0.65-2.27) 0.54	1.48 (1.03-2.11) **0.032**	0.90 (0.36-2.25) 0.81	1.91 (0.81-4.52) 0.14	1.38 (0.81-2.35) 0.24

a Due to the absence of any detected instance in the case group, it was not possible to calculate the corresponding odds ratio (OR) value. P value less than 0.05 were bold. NA, not available.

Detailed results of symptom-organism associations were shown in **Tables 3** and **4**. Proportions of STI-related organisms in cases and controls were shown in **Supplementary Tables 11** and **12**.

## 4 Discussion

Given that different sexually transmitted organisms share the same route of transmission and their infections have similar symptoms. Accurate identification of the causative agents, rather than relying on empiric treatment, can promote responsible antimicrobial use and stewardship. Therefore, targeted screening within higher-risk key population groups can mitigate the socioeconomic burden, and early diagnosis can alleviate patients’ anxiety with multiple clinic visits. Currently, there is a lack of large-scale research in China investigating high-risk populations for diverse STI-related organism infections and their sociodemographic features. Here, we revealed that unmarried status, lower education level, and absence of health insurance coverage were associated with an elevated positivity rate for multiple STI-related organisms. These organisms include not only well-established pathogens like *N. gonorrhoeae*, *C. trachomatis*, and *M. genitalium*, but also HSVs, which are rarely selected as routine screening targets (Karellis et al., 2022; Pereyre et al., 2022) and organisms with controversial pathogenicity, such as *M. hominis* (Taylor-Robinson, 2017; Hughes and Saunders, 2018). Previous studies on the prevalence of sexually transmitted pathogens have primarily been country-based and have found that low- and middle-income countries bear a higher disease burden (Rowley et al., 2019). Shenzhen, where this study was conducted, is located in one of China’s most economically developed regions. Despite this, significant differences were observed in the detection rates of various STI-related microorganisms. These findings indicated that individuals with lower incomes and education levels in large cities also required significant attention and medical resources. The higher prevalence of STIs among single or divorced individuals and those with lower education levels can be attributed to a combination of behavioral and social factors (Nahmias and Nahmias, 2011). The behavioral factors include sexual practices, contraceptive use and health seeking behavior (Pouget, 2017), while the social factors include socioeconomic status, social network and the availability to knowledge about STIs (Wayal et al., 2019).

The prevalence of bacterial sexually transmitted pathogens among women of reproductive age exhibits geographical variability across different regions of the world. Several studies with similar populations to ours (sexually active adult women attending STI clinics) employed molecular tests to obtain and the detection rates of STI pathogens. In an Australian study, *N. gonorrhoeae* and *C. trachomatis* were detected in 1% and 8% of the enrolled women, and the prevalence of *M. genitalium* and *T. vaginalis* were 6% and 0.87% respectively (Latimer et al., 2022). In another epidemiological study of STI pathogens conducted in two cities in southern China, the prevalence of *N. gonorrhoeae*, *C. trachomatis*, *M. genitalium* and *T. vaginalis* were 1.8%, 15.4%, 1.9% and 1.8% among female participants respectively (Kang et al., 2022). In this study, the infection rates of *N. gonorrhoeae*, *C. trachomatis*, *M. genitalium* and *T. vaginalis* among female participants were 1.8%, 10.0%, 2.3% and 2.1%, respectively. Based on the above studies, the prevalence of *C. trachomatis* is the highest, reaching or surpassing 10%, whereas the prevalence of *M. genitalium* exhibits significant variability.

There were 831 male participants with symptoms of urethritis (urethral discharge or irritation), of which 126 (15.2%) were detected with *N. gonorrhoeae* and the remaining 705 N*. gonorrhoeae*-negative males were defined as nongonococcal urethritis (NGU). In this study, the prevalence of *C. trachomatis*, *M. genitalium* and *T. vaginalis* among NGU was 17.2% (N=121), 5.1% (N=36) and 0.6% (N=4) respectively. *N. gonorrhoeae* and *C. trachomatis* are well-accepted etiologic agents of urethritis and in United States, about 5%-20% of urethritis is caused by *N. gonorrhoeae* infection (Bachmann et al., 2015), while *C. trachomatis* infection accounts for the highest proportion of NGU ranging from 15% to 40% (Wetmore et al., 2011). The prevalence of the two agents in our study were similar to those in United States. *M. genitalium* is now recognized as an established etiology of NGU and it is estimated to cause 13% to 31% of NGU cases in the US (Schwebke et al., 2011; Manhart et al., 2013). Furthermore, we also reported a lower detection rate of *T. vaginalis* compared to several published studies (Schwebke et al., 2011; Gaydos et al., 2013; Manhart et al., 2013). The comparatively lower detection rates of *T. vaginalis* and *M. genitalium* among urethritis cases suggest variations in their prevalence among different countries.

We believe that females infected with *M. hominis* deserved further attention. While its involvement in urogenital infections and its impact on reproductive health remain controversial, previous studies have reported associations between bacterial vaginosis (BV) and suggested *M. hominis* might promote the proliferation of other BV-associated bacteria (Taylor-Robinson, 2017; Rumyantseva et al., 2019; Plummer et al., 2021). In our study, detection of *M. hominis* was linked to abnormal vaginal discharge and vaginal itch, which are manifestations of BV, thereby supporting the association between *M. hominis* and BV. BV is associated with an overgrowth of *Gardnerella vaginalis*. Testing *G. vaginalis* and exploring the relationship between its level and *M. hominis* will help further clarify the role of *M. hominis* in BV.

Furthermore, the increased detection of *M. hominis* exhibited a significant correlation with lower education level and the absence of health insurance coverage. This underlines the potential substantial prevalence of *M. hominis* in China, or other populous developing countries. Additionally, we demonstrated a significant association between *M. hominis* and epididymis swelling or pain, which has provided insights into the etiology of epididymitis (Pilatz et al., 2015).

*M. genitalium* has rapidly acquired resistance against recommended treatment, US CDC has escalated it to the antimicrobial resistance threats watch list in 2019 (Centers for Disease Control and Prevention, 2019), but the surveillance data in China were limited. In our study, macrolide-resistant *M. genitalium* were found in 83.9% *M. genitalium*-positive samples, close to the prevalence reported in Nanjing (Li et al., 2020), higher than those reported in the United States (59.1% (Manhart et al., 2023) and 52% (Getman et al., 2023)), Australia (62%) (Sweeney et al., 2019), and Uganda (10.7%) (Melendez et al., 2022). Fluoroquinolone resistance is conferred by mutations in ParC and GyrA. Our study and others in Australia and China reported that less than 10% of *M. genitalium* had GyrA mutations, with all of these cases cooccurring with ParC mutations (Murray et al., 2017; Ke et al., 2020; Murray et al., 2023). Therefore, ParC, especially the Ser83Ile mutation, serves as a valuable marker to guide the clinical use of fluoroquinolones (Sweeney et al., 2022). Based on our study, the prevalence of wild-type ParC and ParC Ser83 were 26.7% and 39.8%, close to those reported in Guangzhou. These data indicate large cities in southern China have a low prevalence of wild-type of ParC, which will result in a higher risk of fluoroquinolone treatment failure. We also found that in participants lack of health insurance, there was a significant rise in the proportion of quinolone-resistant *M. genitalium*. This is likely attributed to patients seeking care from non-formal medical facilities or self-medicating.

HSV is the common cause of viral STI. A study in the United States found no significant differences in the manifestations of genital tract infection caused by HSV-1 and HSV-2 (Bernstein et al., 2013). However, in our study, HSV-1 exhibited a greater correlation with female urogenital symptoms compared to HSV-2. While the association between genital lesions and HSV-2 was only observed in male participants. The study in the United States has demonstrated variations in the epidemiological features of HSV across different age groups and racial populations. Consequently, the prevalence of HSV in China may exhibit distinct characteristics compared to United States. The data presented here will contribute novel evidence regarding the pathogenicity of HSV.

A key strength of this study is the large sample size, comprising 7886 participants, with both men and women. The participants encompassed both asymptomatic and symptomatic individuals presenting with a range of urogenital symptoms. Another strength is the broad screening of STI-related organisms using a highly sensitive mass spectrometry-based method. Finally, we systematically gathered sociodemographic information including marital status, educational attainment, health insurance coverage, income, etc. and these characteristics were found to be correlated with specific STI-related organisms.

One limitation of the study was the incapacity of mass spectrometry-based screening methods to quantify bacterial loads. Previous studies have reported associations between high bacterial loads of *U. urealyticum* and *U. parvum* with nongonococcal urethritis in men (Deguchi et al., 2015; Frolund et al., 2016). Additionally, high bacterial loads of *M. hominis* have been linked to dysbiosis in patients with BV (Cox et al., 2016; Taylor-Robinson, 2017). Therefore, by understanding bacterial loads, we can uncover a more detailed link between infectious organisms and specific symptoms. Through identifying the appropriate cut-off level of bacterial load, the role of infected organisms in patients with urogenital symptoms can be determined. Furthermore, investigating the correlation between sociodemographic characteristics and the bacterial load is a topic meriting exploration. Another limitation of the study was that urethral or cervical swabs were used as the only sample type for STI-related organism screening. Although there have been currently limited researches on the distribution of STI-related organisms across different sample types, Getman et al. found that the distribution of *M. genitalium* from female urogenital tract was highly complex, with diverse phenotypic combinations at different anatomic sites (Getman et al., 2023). Therefore, other STI-related organisms may exhibit distinctive distribution patterns across different sample types.

## 5 Conclusion

This study investigated the prevalence of STI-related organisms in Shenzhen, a representative large city in southern China and revealed a high prevalence of antimicrobial-resistant *M. genitalium*. Unmarried status, lower education level, and absence of health insurance coverage were important sociodemographic factors associated with an elevated positivity rate for multiple STI-related organisms. We found evidence supporting the association between *M. hominis* and BV or epididymitis. *M. hominis* exhibited a significant correlation with lower education level and the absence of health insurance coverage, which underlines the potential substantial prevalence of *M. hominis* in China. For HSV infection, we found HSV-1 exhibited a greater correlation with female urogenital symptoms. Additionally, we found a possible association between quinolone-resistant *M. genitalium* and lack of health insurance. These findings offer valuable insights into higher-risk key population groups, aiding the development of screening strategies and personalized guidelines for *M. genitalium* treatment.

## Data availability statement

The raw data supporting the conclusions of this article will be made available by the authors, without undue reservation.

## Ethics statement

This study was performed in accordance with the recommendations of the national ethics regulations and approved by the Medical Ethics Committee of Shenzhen Center for Chronic Disease Control (20180301). All the participants provided written informed consent.

## Author contributions

FFW: Conceptualization, Data curation, Formal Analysis, Project administration, Resources, Supervision, Writing – original draft, Writing – review & editing. CZ: Data curation, Formal Analysis, Investigation, Methodology, Writing – original draft, Writing – review & editing. LX: Methodology, Validation, Writing – review & editing. YML: Methodology, Validation, Writing – review & editing. YZ: Methodology, Resources, Writing – review & editing. YZL: Methodology, Resources, Writing – review & editing. YC: Conceptualization, Data curation, Project administration, Resources, Supervision, Validation, Writing – review & editing. JP: Conceptualization, Funding acquisition, Investigation, Project administration, Supervision, Validation, Writing – original draft, Writing – review & editing.
