# Supplementary material for: Etiological, sociodemographic and clinical characteristics of sexually transmitted infections and M. genitalium resistance in Shenzhen: a multicenter cross-sectional study in China
Source: Front Cell Infect Microbiol. 2024 Jul 25;14:1407124. doi: 10.3389/fcimb.2024.1407124 (PMC11308211; doi:10.3389/fcimb.2024.1407124)
Supplement: Supplementary file 1 [file Table_1.docx]

Supplementary Materials

Table of contents

[Table S1. Numbers and proportions of samples collected by each hospital 2](#_Toc153229495)

[Table S2. Primers used for amplification of the macrolide and fluoroquinolone resistance-determining region located in 23S rRNA, the *parC* and *gyrA* gene. 3](#_Toc153229496)

[Table S3. Socio-demographic characteristics of eligible participants 4](#_Toc153229497)

[Table S4. Clinical signs and symptoms of eligible participants 6](#_Toc153229498)

[Table S5. Detection of nine STI-related organisms 7](#_Toc153229499)

[Table S6. Univariable analysis of socio-demographic factors associated with *N. gonorrhoeae*, *C. trachomatis* and *T*. vaginalis. 8](#_Toc153229500)

[Table S7. Univariable analysis of socio-demographic factors associated with *M. genitalium*, HSV-1, HSV-2. 10](#_Toc153229501)

[Table S8. Univariable analysis of socio-demographic factors associated with *M. hominis, U. parvum* and *U. urealyticum.* 12](#_Toc153229502)

[Table S9. Multivariable analysis of sociodemographic factors associated with *N. gonorrhoeae* and *C. trachomatis* 14](#_Toc153229503)

[Table S10. Multivariable analysis of sociodemographic factors associated with STI-related organisms 15](#_Toc153229504)

[Table S11. Proportions of STI-related microorganisms in male cases and controls 17](#_Toc153229505)

[Table S12. Proportions of STI-related microorganisms in female cases and controls 18](#_Toc153229506)

Table S1. Numbers and proportions of samples collected by each hospital

| **Table S1. Numbers and proportions of samples collected by each hospital** | | | |
| --- | --- | --- | --- |
| Hospital | No. (%) | Hospital | No. (%) |
| **Luohu District** |  | **Longgang District** |  |
| Shenzhen People's Hospital | 51 (0.6) | Shenzhen Longgang District Maternity and Child Healthcare Hospital | 551 (6.9) |
| Shenzhen Center for Chronic Disease Control | 122 (1.5) | Fifth People’s Hospital of Longgang District Shenzhen | 272 (3.4) |
| Shenzhen Luohu Maternity and Child Healthcare Hospital | 630 (7.9) | The Third People’s Hospital of Longgang District Shenzhen | 310 (3.9) |
| Shenzhen Luohu Hospital of Traditional Chinese Medicine | 205 (2.6) | Longgang District People’s Hospital of Shenzhen | 380 (4.8) |
| Shenzhen Luohu Hospital Group Luohu People’s Hospital | 466 (5.8) | **Nanshan District** |  |
| **Baoan District** |  | Shenzhen Nanshan Maternity and Child Healthcare Hospital | 213 (2.7) |
| Shenzhen FuYong People's Hospital | 641 (8.0) | Shenzhen Nanshan Xili People’s Hospital | 323 (4.0) |
| Shenzhen Baoan Maternal and Child Health Hospital | 665 (8.3) | Shenzhen Nanshan Hospital | 543 (6.8) |
| Shenzhen Baoan People's Hospital | 351 (4.4) | Shenzhen Nanshan Shekou People’s Hospital | 431 (5.4) |
| Shenzhen Baoan Shajing People’s Hospital | 675 (8.4) | **Longhua District** |  |
| **Yantian District** |  | Shenzhen Longhua District Maternity and Child Healthcare Hospital | 120 (1.5) |
| Yantian District Maternity and Child Healthcare Hospital | 118 (1.5) | Shenzhen Longhua District Central Hospital | 294 (3.7) |
| Shenzhen Yantian District People’s Hospital | 252 (3.2) | Shenzhen Longhua People’s Hospital | 377 (4.7) |

Table S2. Primers used for amplification of the macrolide and fluoroquinolone resistance-determining region located in 23S rRNA, the *parC* and *gyrA* gene.

| **Table S2. Primers used for amplification of the macrolide and fluoroquinolone resistance-determining region located in 23S rRNA, the *parC* and *gyrA* gene.** | | | | |
| --- | --- | --- | --- | --- |
| **Gene and amplified regions** | **Primer name** | **Sequences** | **Primer name** | **Sequences** |
| 23S rRNA (2031-2258) |  |  |  |  |
|  | MG-23S-1972F  (outer primer) | CGTCCCGCTTGAATGGTGTA | MG-23S-2299R  (outer primer) | TGCGCCTCCGTTACCTTTTA |
|  | MG-23S-2031F  (inner primer) | CCAGGTACGGGTGAAGACAC | MG-23S-2258R  (inner primer) | ACTGCCCACCTAACACTGTC |
| *gyrA* (171-400) |  |  |  |  |
|  | MG-gyrA-127F  (outer primer) | GCTCGTGCTTTACCTGATGC | MG-gyrA-522R  (outer primer) | AGCTGCTGGTAGAACAGTTGG |
|  | MG-gyrA-171F  (inner primer) | TCGTCGTGTTCTTTATGGTGCT | MG-gyrA-400R  (inner primer) | AACGTTGTGCAGCAGGTCTA |
| *parC* (202-382) |  |  |  |  |
|  | MG-parC-167F  (outer primer) | GCTTAAAACCCACCACTYCCT | MG-parC-483R  (outer primer) | GGTTGGTAAAACCGTTGGTTCT |
|  | MG-parC-202F  (inner primer) | CGTGCTGTTGGGGAGATCAT | MG-parC-382R  (inner primer) | TTAAGCGGGTTTCTGTGTAACG |

Table S3. Socio-demographic characteristics of eligible participants

| **Table S3** **Socio-demographic characteristics of eligible participants^a^** | | |
| --- | --- | --- |
| **Socio-demographic characteristics** | **No. (%) of** | |
|  | **Male (n=2466)** | **Female (n=5420)** |
| Age in years [median (IQR)] | 31 (27-37) | 31 (27-36) |
| Missing | 5 (0.2) | 35 (0.6) |
| Marital status |  |  |
| Currently married | 1603 (65.0) | 4274 (78.9) |
| Single or divorced | 858 (34.8) | 1102 (20.3) |
| Missing | 5 (0.2) | 44 (0.8) |
| Education |  |  |
| Primary or junior high school | 667 (27.0) | 1823 (33.6) |
| Senior hs or vocational hs | 757 (30.7) | 1531 (28.2) |
| College or graduate | 1014 (41.1) | 1998 (36.9) |
| Missing | 28 (1.1) | 68 (1.3) |
| Health insurance coverage |  |  |
| Yes | 1481 (60.1) | 3358 (62.0) |
| No | 968 (39.3) | 1991 (36.7) |
| Missing | 17 (0.7) | 71 (1.3) |
| Monthly income |  |  |
| Less than $500 | 225 (9.1) | 1088 (20.1) |
| More than $500 | 2113 (85.7) | 4057 (74.9) |
| Missing | 128 (5.2) | 275 (5.1) |
| Sexual orientation |  |  |
| Heterosexual | 2382 (96.6) | 5242 (96.7) |
| Homosexual or bisexual | 58 (2.4) | 45 (0.8) |
| Missing | 26 (1.1) | 133 (2.5) |
| Casual sex partner in past 3 months |  |  |
| No | 1132 (45.9) | 3808 (70.3) |
| Yes | 1308 (53.0) | 1531 (28.2) |
| Missing | 26 (1.1) | 81 (1.5) |
| Residency in Shenzhen |  |  |
| Yes | 537 (21.8) | 1471 (27.1) |
| No | 1900 (77) | 3839 (70.8) |
| Missing | 29 (1.2) | 110 (2.0) |
| Living time in Shenzhen |  |  |
| Less than 6 months | 140 (5.7) | 426 (7.9) |
| More than 6 months | 2297 (93.1) | 4902 (90.4) |
| Missing | 29 (1.2) | 92 (1.7) |
| Occupation |  |  |
| Manual workers | 877 (35.6) | 1084 (20) |
| Entertainment/catering industry | 127 (5.2) | 251 (4.6) |
| Commercial industry | 498 (20.2) | 720 (13.3) |
| Government staff | 540 (21.9) | 1278 (23.6) |
| Housewife/househusband | 1 (0) | 912 (16.8) |
| Unemployed | 39 (1.6) | 239 (4.4) |
| Others | 373 (15.1) | 891 (16.4) |
| Missing | 11 (0.4) | 45 (0.8) |
| ^a^Abbreviations: IQR, interquartile range | | |

Table S4. Clinical signs and symptoms of eligible participants

| **Table S4** **Clinical signs and symptoms of eligible participants** | | | |
| --- | --- | --- | --- |
| **Clinical signs and symptoms of male** | **No. (%) of**  **Male**  **n=2466** | **Clinical signs and symptoms of female** | **No. (%) of**  **Female**  **n=5420** |
| No symptoms (Control) | 1176 (47.7) | No symptoms (Control) | 1966 (36.3) |
| Urethral serous discharge | 183 (7.4) | Abnormal vaginal discharge | 2917 (53.8) |
| Urethral purulent discharge | 161 (6.5) | Mucopurulent cervicitis | 260 (4.8) |
| Scrotum swelling or pain | 68 (2.8) | Lower abdominal pain | 492 (9.1) |
| Epididymis swelling or pain | 35 (1.4) | Vaginal itch | 79 (1.5) |
| Balanoposthitis | 74 (3.0) |  |  |
| Urethral burning or irritation | 619 (25.1) | Urethral burning or irritation | 251 (4.6) |
| Genital or perianal blisters | 72 (2.9) | Genital or perianal blisters | 41 (0.8) |
| Genital or perianal ulcers | 52 (2.1) | Genital or perianal ulcers | 34 (0.6) |
| Genital or perianal warts | 182 (7.4) | Genital or perianal warts | 100 (1.8) |

Table S5. Detection of nine STI-related organisms

| **Table S5** **Detection of nine STI-related organisms^a^** | | | |
| --- | --- | --- | --- |
| **Microorganisms** | **No. (%, 95% CI) of**  **Male**  **n=2466** | **No. (%, 95%CI) of**  **Female**  **n=5420** | **P value**^b^ |
| *N. gonorrhoeae* | 221 (9.0, 7.9-10.2) | 98 (1.8, 1.5-2.2) | **<.001** |
| *C. trachomatis* | 287 (11.6, 10.4-13.0) | 542 (10.0, 9.2-10.8) | **.004** |
| *T. vaginalis* | 14 (0.6, 0.3-1.0) | 111 (2.1, 1.7-2.5) | **<.001** |
| *M. genitalium* | 70 (2.8, 2.3-3.6) | 122 (2.3, 1.9-2.7) | .134 |
| HSV-1 | 43 (1.7, 1.3-2.3) | 85 (1.6, 1.3-1.9) | .565 |
| HSV-2 | 31 (1.3, 0.9-1.8) | 77 (1.4, 1.1-1.8) | .603 |
| *M. hominis* | 162 (6.6, 5.7-7.6) | 758 (14, 13.1-14.9) | **<.001** |
| *U. parvum* | 427 (17.3, 15.9-18.9) | 2774 (51.2, 49.9-52.5) | **<.001** |
| *U. urealyticum* | 299 (12.1, 10.9-13.5) | 776 (14.3, 13.4-15.3) | **.009** |
| No microorganisms detected | 1371 (55.6, 53.6-57.6) | 1848 (34.1, 32.9-35.4) | **<.001** |
| ^a^Abbreviations: CI, confidence interval.  ^b^P-values <0.05 are bolded to indicate that the difference of detection rates between male and female are statistically significant. | | | |

Table S6. Univariable analysis of socio-demographic factors associated with *N. gonorrhoeae*, *C. trachomatis* and *T*. vaginalis.

| **Table S6** **Univariable analysis of socio-demographic factors associated with *N. gonorrhoeae*, *C. trachomatis* and *T. vaginalis*.** | | | | | | | | | | | | |
| --- | --- | --- | --- | --- | --- | --- | --- | --- | --- | --- | --- | --- |
| Sociodemographic factors | *N. gonorrhoeae* | | | | *C. trachomatis* | | | | *T. vaginalis* | | | |
|  | Negative  N=7567 | Positive  N=319 | OR (95% CI) | P | Negative  N=7057 | Positive  N=829 | OR (95% CI) | P | Negative  N=7761 | Positive  N=125 | OR (95% CI) | P |
| Gender |  |  |  |  |  |  |  |  |  |  |  |  |
| Male | 2245 | 221 | 1 |  | 2179 | 287 | 1 |  | 2452 | 14 | 1 |  |
| Female | 5322 | 98 | 0.19 (0.15-0.24) | **<.001** | 4878 | 542 | 0.84 (0.73-0.98) | **.028** | 5309 | 111 | 3.66 (2.10-6.40) | **<.001** |
| Age (median, IQR) | 31 (9) | 29.5(11) | 0.98 (0.96-1.00) | **.009** | 31 (10) | 29 (10) | 0.97 (0.96-0.98) | **<.001** | 31 (9) | 30 (14) | 1.00 (0.97-1.02) | .695 |
| Marital status |  |  |  |  |  |  |  |  |  |  |  |  |
| Currently married | 5685 | 192 | 1 |  | 5350 | 527 | 1 |  | 5792 | 85 | 1 |  |
| Single or divorced | 1834 | 126 | 2.03 (1.62-2.56) | **<.001** | 1661 | 299 | 1.83 (1.57-2.13) | **<.001** | 1922 | 38 | 1.35 (0.92-1.98) | .130 |
| Education |  |  |  |  |  |  |  |  |  |  |  |  |
| Primary or junior high school | 2409 | 81 | 1 |  | 2197 | 293 | 1 |  | 2438 | 52 | 1 |  |
| Senior hs or vocational hs | 2185 | 103 | 1.40 (1.04-1.89) | **.026** | 2030 | 258 | 0.95 (0.80-1.14) | .596 | 2254 | 34 | 0.71 (0.46-1.09) | .119 |
| College or graduate | 2882 | 130 | 1.34 (1.01-1.78) | **.042** | 2747 | 265 | 0.72 (0.61-0.86) | **<.001** | 2974 | 38 | 0.60 (0.39-0.91) | **.017** |
| Health insurance coverage |  |  |  |  |  |  |  |  |  |  |  |  |
| Yes | 4663 | 176 | 1 |  | 4401 | 438 | 1 |  | 4778 | 61 | 1 |  |
| No | 2821 | 138 | 1.30 (1.03-1.63) | **.026** | 2578 | 381 | 1.49 (1.28-1.72) | **<.001** | 2898 | 61 | 1.65 (1.15-2.36) | **.006** |
| Monthly income |  |  |  |  |  |  |  |  |  |  |  |  |
| Less than $500 | 1281 | 32 | 1 |  | 1164 | 149 | 1 |  | 1278 | 35 | 1 |  |
| More than $500 | 5899 | 271 | 1.84 (1.37-2.67) | **.001** | 5523 | 647 | 0.92 (0.76-1.11) | .358 | 6087 | 83 | 0.50 (0.33-0.74) | **.001** |
| Sexual orientation |  |  |  |  |  |  |  |  |  |  |  |  |
| Heterosexual | 7313 | 311 | 1 |  | 6823 | 801 | 1 |  | 7504 | 120 | 1 |  |
| Homosexual or bisexual | 101 | 2 | 0.47 (0.11-1.90) | .286 | 96 | 7 | 0.62 (0.29-1.34) | .226 | 103 | 0 | NA^a^ | NA^a^ |
| Casual sex partner in past 3 months |  |  |  |  |  |  |  |  |  |  |  |  |
| No | 4804 | 136 | 1 |  | 4440 | 500 | 1 |  | 4863 | 77 | 1 |  |
| Yes | 2657 | 182 | 2.42 (1.93-3.04) | **<.001** | 2523 | 316 | 1.11 (0.96-1.29) | .162 | 2794 | 45 | 1.02 (0.70-1.47) | .928 |
| Residency in Shenzhen |  |  |  |  |  |  |  |  |  |  |  |  |
| Yes | 1939 | 69 | 1 |  | 1842 | 166 | 1 |  | 1981 | 27 | 1 |  |
| No | 5492 | 247 | 1.26 (0.96-1.66) | **.091** | 5092 | 647 | 1.41 (1.18-1.69) | **<.001** | 5646 | 93 | 1.21 (0.79-1.86) | .390 |
| Living time in Shenzhen |  |  |  |  |  |  |  |  |  |  |  |  |
| Less than 6 months | 544 | 22 | 1 |  | 498 | 68 | 1 |  | 549 | 17 | 1 |  |
| More than 6 months | 6905 | 294 | 1.05 (0.68-1.64) | .819 | 6448 | 751 | 0.85 (0.66-1.11) | .238 | 7092 | 107 | 0.49 (0.29-0.82) | **.007** |
| Occupation |  |  |  |  |  |  |  |  |  |  |  |  |
| Commercial industry | 1152 | 66 | 1.39 (0.72-2.67) | .322 | 1082 | 136 | 0.90 (0.60-1.35) | .614 | 1195 | 23 | 0.75 (0.32-1.75) | .501 |
| Entertainment/  catering industry | 365 | 13 | 0.87 (0.38-1.96) | .727 | 333 | 45 | 0.97 (0.60-1.56) | .899 | 369 | 9 | 0.94 (0.35-2.57) | .910 |
| Government staff | 1742 | 76 | 1.06 (0.56-2.02) | .862 | 1661 | 157 | 0.68 (0.46-1.01) | **.054** | 1794 | 24 | 0.52 (0.22-1.21) | .130 |
| Housewife/  househusband | 900 | 13 | 0.35 (0.16-0.79) | **.012** | 820 | 93 | 0.81 (0.54-1.24) | .334 | 898 | 15 | 0.65 (0.26-1.60) | .346 |
| Manual workers | 1860 | 101 | 1.32 (0.70-2.49) | .394 | 1732 | 229 | 0.95 (0.65-1.39) | .789 | 1938 | 23 | 0.46(0.20-1.08) | **.075** |
| Others | 1226 | 38 | 0.75 (0.38-1.49) | .415 | 1133 | 131 | 0.83 (0.56-1.24) | .363 | 1240 | 24 | 0.75(0.32-1.76) | .507 |
| Unemployed | 267 | 11 | 1 |  | 244 | 34 | 1 |  | 2271 | 7 | 1 |  |
| P values < 0.1 are bolded to indicate that the corresponding factors were included in the multivariable analyses.  ^a^ Due to the absence of any detected instance in the homosexual or bisexual group, it was not possible to calculate the corresponding odds ratio (OR) value. | | | | | | | | | | | | |

Table S7. Univariable analysis of socio-demographic factors associated with *M. genitalium*, HSV-1, HSV-2.

| **Table S7** **Univariable analysis of socio-demographic factors associated with *M. genitalium*, HSV-1, HSV-2.** | | | | | | | | | | | | |
| --- | --- | --- | --- | --- | --- | --- | --- | --- | --- | --- | --- | --- |
| Sociodemographic factors | *M. genitalium* | | | | HSV-1 | | | | HSV-2 | | | |
|  | Negative  N=7694 | Positive  N=192 | OR (95% CI) | P | Negative  N=7758 | Positive  N=128 | OR (95% CI) | P | Negative  N=7778 | Positive  N=108 | OR (95% CI) | P |
| Gender |  |  |  |  |  |  |  |  |  |  |  |  |
| Male | 2396 | 70 | 1 |  | 2423 | 43 | 1 |  | 2435 | 31 | 1 |  |
| Female | 5298 | 112 | 0.79 (0.59-1.06) | .117 | 5335 | 85 | 0.90 (0.62-1.30) | .568 | 5343 | 77 | 1.13 (0.74-1.72) | .563 |
| Age (median, IQR) | 31 (9) | 29 (10) | 0.95 (0.93-0.97) | **<.001** | 31 (9) | 30(10.5) | 1.00 (0.97-1.02) | .718 | 31 (9) | 32 (14) | 1.02 (0.99-1.04) | .185 |
| Marital status |  |  |  |  |  |  |  |  |  |  |  |  |
| Currently married | 5763 | 114 | 1 |  | 5792 | 85 | 1 |  | 5809 | 68 | 1 |  |
| Single or divorced | 1882 | 78 | 2.10 (1.56-2.81) | **<.001** | 1917 | 43 | 1.53 (1.06-2.21) | **.025** | 1921 | 39 | 1.73 (1.17-2.58) | **.007** |
| Education |  |  |  |  |  |  |  |  |  |  |  |  |
| Primary or junior high school | 2421 | 69 | 1 |  | 2451 | 39 | 1 |  | 2451 | 39 | 1 |  |
| Senior hs or vocational hs | 2230 | 58 | 0.91 (0.64-1.30) | .612 | 2243 | 45 | 1.26 (0.82-1.94) | .294 | 2244 | 44 | 1.23 (0.80-1.90) | .346 |
| College or graduate | 2948 | 64 | 0.76 (0.54-1.08) | .121 | 2969 | 43 | 0.91 (0.59-1.41) | .673 | 2989 | 23 | 0.48 (0.29-0.81) | **.006** |
| Health insurance coverage |  |  |  |  |  |  |  |  |  |  |  |  |
| Yes | 4737 | 102 | 1 |  | 4771 | 68 | 1 |  | 4783 | 56 | 1 |  |
| No | 2869 | 90 | 1.46 (1.09-1.94) | **.010** | 2900 | 59 | 1.43 (1.00-2.03) | **.047** | 2908 | 51 | 1.50 (1.02-2.20) | **.038** |
| Monthly income |  |  |  |  |  |  |  |  |  |  |  |  |
| Less than $500 | 1276 | 37 | 1 |  | 1289 | 24 | 1 |  | 1290 | 23 | 1 |  |
| More than $500 | 6025 | 145 | 0.83 (0.58-1.20) | .318 | 6071 | 99 | 0.88 (0.56-1.37) | .564 | 6090 | 80 | 0.74 (0.46-1.18) | .200 |
| Sexual orientation |  |  |  |  |  |  |  |  |  |  |  |  |
| Heterosexual | 7441 | 183 | 1 |  | 7500 | 124 | 1 |  | 7518 | 106 | 1 |  |
| Homosexual or bisexual | 101 | 2 | 0.81 (0.20-3.29) | .763 | 101 | 2 | 1.20 (0.29-4.91) | .802 | 102 | 1 | 0.70 (0.10-5.03) | .719 |
| Casual sex partner in past 3 months |  |  |  |  |  |  |  |  |  |  |  |  |
| No | 4834 | 106 | 1 |  | 4862 | 78 | 1 |  | 4873 | 67 | 1 |  |
| Yes | 2756 | 83 | 1.37 (1.03-1.84) | **.033** | 2791 | 48 | 1.07 (0.75-1.54) | .707 | 2799 | 40 | 1.04 (0.70-1.54) | .848 |
| Residency in Shenzhen |  |  |  |  |  |  |  |  |  |  |  |  |
| Yes | 1978 | 30 | 1 |  | 1971 | 37 | 1 |  | 1985 | 23 | 1 |  |
| No | 5581 | 158 | 1.87 (1.26-2.77) | **.002** | 5651 | 88 | 0.83 (0.56-1.22) | .344 | 5657 | 82 | 1.25 (0.79-1.99) | .345 |
| Living time in Shenzhen |  |  |  |  |  |  |  |  |  |  |  |  |
| Less than 6 months | 544 | 22 | 1 |  | 555 | 11 | 1 |  | 560 | 6 | 1 |  |
| More than 6 months | 7031 | 168 | 0.59 (0.38-0.93) | **.023** | 7085 | 114 | 0.81 (0.44-1.52) | .513 | 7103 | 96 | 1.26 (0.55-2.89) | .583 |
| Occupation |  |  |  |  |  |  |  |  |  |  |  |  |
| Commercial industry | 1191 | 27 | 0.68 (0.32-1.46) | .319 | 1201 | 17 | 0.48 (0.20-1.12) | **.089** | 1200 | 18 | 1.03 (0.35-3.06) | .961 |
| Entertainment/  catering industry | 356 | 22 | 1.85 (0.84-4.08) | .129 | 367 | 11 | 1.01 (0.40-2.55) | .981 | 367 | 11 | 2.05 (0.45-6.52) | .222 |
| Government staff | 1788 | 30 | 0.50 (0.24-1.07) | **.074** | 1795 | 23 | 0.43 (0.19-0.98) | **.044** | 1807 | 11 | 0.42 (0.13-1.32) | .137 |
| Housewife/  househusband | 896 | 17 | 0.57 (0.25-1.29) | .175 | 895 | 18 | 0.68 (0.29-1.58) | .368 | 898 | 15 | 1.14 (0.38-3.48) | .812 |
| Manual workers | 1907 | 54 | 0.85 (0.41-1.73) | .648 | 1926 | 35 | 0.61 (0.28-1.34) | .218 | 1931 | 30 | 1.06 (0.37-3.04) | .908 |
| Others | 1234 | 30 | 0.73 (0.31-1.55) | .408 | 1249 | 15 | 0.41 (0.17-0.97) | **.041** | 1246 | 18 | 0.99 (0.33-2.95) | .985 |
| Unemployed | 269 | 9 | 1 |  | 270 | 8 | 1 |  | 274 | 4 | 1 |  |
| P values < 0.1 are bolded to indicate that the corresponding factors were included in the multivariable analyses. | | | | | | | | | | | | |

Table S8. Univariable analysis of socio-demographic factors associated with *M. hominis, U. parvum* and *U. urealyticum.*

| **Table S8** **Univariable analysis of socio-demographic factors associated with *M. hominis, U. parvum* and *U. urealyticum.*** | | | | | | | | | | | | |
| --- | --- | --- | --- | --- | --- | --- | --- | --- | --- | --- | --- | --- |
| Sociodemographic factors | *M. hominis* | | | | *U. parvum* | | | | *U. urealyticum* | | | |
|  | Negative  N=6966 | Positive  N=920 | OR (95% CI) | P | Negative  N=4685 | Positive  N=3201 | OR (95% CI) | P | Negative  N=6811 | Positive  N=1075 | OR (95% CI) | P |
| Gender |  |  |  |  |  |  |  |  |  |  |  |  |
| Male | 2304 | 162 | 1 |  | 2039 | 427 | 1 |  | 2167 | 299 | 1 |  |
| Female | 4662 | 758 | 2.31 (1.94-2.76) | **<.001** | 2646 | 2774 | 5.01 (4.45-5.63) | **<.001** | 4644 | 776 | 1.21 (1.05-1.40) | **.009** |
| Age (median, IQR) | 31 (9) | 31 (10) | 1.00 (0.99-1.01) | .392 | 31 (10) | 31 (9) | 0.99 (0.99-1.00) | **.032** | 31 (9) | 30 (10) | 0.98 (0.97-0.99) | **<.001** |
| Marital status |  |  |  |  |  |  |  |  |  |  |  |  |
| Currently married | 5220 | 657 | 1 |  | 3516 | 2361 | 1 |  | 5117 | 760 | 1 |  |
| Single or divorced | 1702 | 258 | 1.20 (1.03-1.41) | **.018** | 1146 | 814 | 1.06 (0.95-1.17) | .289 | 1651 | 309 | 1.26 (1.09-1.45) | **.002** |
| Education |  |  |  |  |  |  |  |  |  |  |  |  |
| Primary or junior high school | 2126 | 364 | 1 |  | 1477 | 1013 | 1 |  | 2111 | 379 | 1 |  |
| Senior hs or vocational hs | 2034 | 254 | 0.73 (0.61-0.87) | **<.001** | 1347 | 941 | 1.02 (0.91-1.14) | .755 | 1969 | 319 | 0.90 (0.77-1.06) | .211 |
| College or graduate | 2725 | 287 | 0.62 (0.52-0.73) | **<.001** | 1803 | 1209 | 0.98 (0.88-1.09) | .683 | 2648 | 364 | 0.77 (0.66-0.89) | **.001** |
| Health insurance coverage |  |  |  |  |  |  |  |  |  |  |  |  |
| Yes | 4336 | 503 | 1 |  | 4771 | 68 | 1 |  | 4783 | 56 | 1 |  |
| No | 2556 | 403 | 1.36 (1.18-1.56) | **<.001** | 2900 | 59 | 1.08 (0.99-1.19) | **.091** | 2908 | 51 | 1.32 (1.16-1.51) | **<.001** |
| Monthly income |  |  |  |  |  |  |  |  |  |  |  |  |
| Less than $500 | 1133 | 180 | 1 |  | 764 | 549 | 1 |  | 1104 | 209 | 1 |  |
| More than $500 | 5479 | 691 | 0.79 (0.67-0.95) | **.010** | 3677 | 2493 | 0.94 (0.84-1.07) | .346 | 5352 | 818 | 0.81 (0.68-0.95) | **.011** |
| Sexual orientation |  |  |  |  |  |  |  |  |  |  |  |  |
| Heterosexual | 6736 | 888 | 1 |  | 4538 | 3086 | 1 |  | 6586 | 1038 | 1 |  |
| Homosexual or bisexual | 95 | 8 | 0.64 (0.31-1.32) | .226 | 74 | 29 | 0.58 (0.37-0.89) | **.012** | 88 | 15 | 1.08 (0.62-1.88) | .781 |
| Casual sex partner in past 3 months |  |  |  |  |  |  |  |  |  |  |  |  |
| No | 4364 | 576 | 1 |  | 2837 | 2103 | 1 |  | 4275 | 665 | 1 |  |
| Yes | 2511 | 328 | 0.99 (0.86-1.14) | .888 | 1792 | 1047 | 0.79 (0.72-0.87) | **<.001** | 2444 | 395 | 1.04 (0.91-1.19) | .576 |
| Residency in Shenzhen |  |  |  |  |  |  |  |  |  |  |  |  |
| Yes | 1807 | 201 | 1 |  | 1172 | 836 | 1 |  | 1780 | 228 | 1 |  |
| No | 5040 | 699 | 1.25 (1.06-1.47) | **.009** | 3438 | 2301 | 0.94 (0.85-1.04) | .227 | 4910 | 829 | 1.32 (1.13-1.54) | **.001** |
| Living time in Shenzhen |  |  |  |  |  |  |  |  |  |  |  |  |
| Less than 6 months | 474 | 92 | 1 |  | 327 | 239 | 1 |  | 478 | 88 | 1 |  |
| More than 6 months | 6382 | 817 | 0.66 (0.52-0.83) | **.001** | 4283 | 2916 | 0.93 (0.78-1.11) | .422 | 6223 | 976 | 0.85 (0.67-1.08) | .185 |
| Occupation |  |  |  |  |  |  |  |  |  |  |  |  |
| Commercial industry | 1081 | 137 | 0.83 (0.56-1.22) | .334 | 739 | 479 | 0.75 (0.58-0.97) | **.031** | 1043 | 175 | 1.00 (0.69-1.45) | .993 |
| Entertainment/  catering industry | 322 | 56 | 1.13 (0.72-1.77) | .585 | 213 | 165 | 0.90 (0.66-1.22) | .484 | 326 | 52 | 0.95 (0.61-1.48) | .818 |
| Government staff | 1640 | 178 | 0.71 (0.48-1.03) | **.073** | 1061 | 757 | 0.82 (0.64-1.06) | .135 | 1625 | 193 | 0.71 (0.49-1.02) | **.063** |
| Housewife/  househusband | 787 | 126 | 1.04 (0.70-1.55) | .835 | 484 | 429 | 1.02 (0.78-1.34) | .864 | 795 | 118 | 0.88 (0.60-1.30) | .529 |
| Manual workers | 1729 | 232 | 0.87 (0.60-1.27) | .478 | 1297 | 664 | 0.59 (0.46-0.76) | **<.001** | 1655 | 306 | 1.10 (0.77-1.57) | .600 |
| Others | 1118 | 146 | 0.85 (0.58-1.25) | .412 | 711 | 553 | 0.90 (0.69-1.17) | .420 | 1082 | 182 | 1.00 (0.69-1.45) | .996 |
| Unemployed | 241 | 37 | 1 |  | 149 | 129 | 1 |  | 238 | 40 | 1 |  |
| P values < 0.1 are bolded to indicate that the corresponding factors were included in the multivariable analyses. | | | | | | | | | | | | |

Table S9. Multivariable analysis of sociodemographic factors associated with *N. gonorrhoeae* and *C. trachomatis*

| **Table S9** **Multivariable analysis of sociodemographic factors associated with *N. gonorrhoeae* and *C. trachomatis*** | | | | |
| --- | --- | --- | --- | --- |
| **Sociodemographic factors** | ***N. gonorrhoeae*** | | ***C. trachomatis*** | |
|  | aOR (95%CI) | P value | aOR (95%CI) | P value |
| Female | 0.24 (0.18-0.32) | **<.001** | 0.88 (0.75-1.05) | .156 |
| Age | 0.99 (0.97-1.01) | .206 | 0.98 (0.97-0.99) | **.001** |
| Single or divorced | 1.35 (1.00-1.83) | **.047** | 1.54 (1.27-1.86) | **<0.001** |
| Education |  |  |  |  |
| Primary or junior high school | 1 |  | 1 |  |
| Senior or vocational high school | 1.19 (0.86-1.65) | .298 | 0.90 (0.74-1.08) | .255 |
| College or graduate | 1.25 (0.87-1.80) | .235 | 0.73 (0.59-0.91) | **.006** |
| No insurance coverage | 1.17 (0.90-1.54) | .243 | 1.17 (0.99-1.39) | .059 |
| Monthly income more than $500 | 1.29 (0.87-1.92) | .211 |  |  |
| Having casual sex partner in last 3 months | 1.70 (1.32-2.18) | **<.001** |  |  |
| Residency in Shenzhen | 1.03 (0.74-1.43) | .878 | 1.03 (0.84-1.27) | .775 |
| Occupation |  |  |  |  |
| Commercial industry | 0.65 (0.33-1.31) | .229 | 0.98 (0.65-1.50) | .942 |
| Entertainment/catering industry | 0.35 (0.14-0.87) | **.023** | 0.83 (0.51-1.37) | .477 |
| Government staff | 0.62 (0.31-1.25) | .181 | 0.84 (0.55-1.29) | .424 |
| Housewife/househusband | 0.46 (0.19-1.12) | .086 | 1.06 (0.68-1.65) | .794 |
| Manual workers | 0.59 (0.30-1.16) | .129 | 0.93 (0.62-1.40) | .724 |
| Others | 0.46 (0.22-0.94) | **.032** | 0.85 (0.56-1.30) | .454 |
| Unemployed | 1 |  | 1 |  |

Table S10. Multivariable analysis of sociodemographic factors associated with STI-related organisms

| **Table S10** **Multivariable analysis of sociodemographic factors associated with STI-related organisms.** | | | | | | | |
| --- | --- | --- | --- | --- | --- | --- | --- |
| **Factors**  **aOR (95%CI)**  **P value** | ***T. vaginalis*** | ***M. genitalium*** | **HSV-1** | **HSV-2** | ***M. hominis*** | ***U. parvum*** | ***U. urealyticum*** |
| Residency in Shenzhen |  | 1.45 (0.94-2.24)  .091 |  |  | 0.97 (0.80-1.19)  .774 |  | 1.07 (0.89-1.29)  .486 |
| Living in Shenzhen > 6 months | 0.72 (0.41-1.28)  .267 | 0.80 (0.49-1.30)  .362 |  |  | 0.84 (0.64-1.09)  .190 |  |  |
| Homosexual or bisexual |  |  |  |  |  | 0.80 (0.50-1.27)  .341 |  |
| Occupation |  |  |  |  |  |  |  |
| Commercial industry | 1.00 (0.41-2.44)  .997 | 0.74 (0.33-1.66)  .458 | 0.48 (0.20-1.13)  .094 |  | 1.05 (0.69-1.61)  .804 | 1.08 (0.81-1.43)  .611 | 1.12 (0.75-1.67)  .590 |
| Entertainment/catering industry | 0.96 (0.34-2.71)  .940 | 1.60 (0.69-3.72)  .278 | 0.94 (0.37-2.37)  .888 |  | 1.14 (0.70-1.85)  .599 | 1.10 (0.78-1.54)  .589 | 0.88 (0.54-1.42)  .590 |
| Government staff | 0.86 (0.35-2.13)  .751 | 0.60 (0.27-1.35)  .219 | 0.47 (0.20-1.08)  .074 |  | 0.92 (0.60-1.40)  .689 | 1.02 (0.77-1.34)  .914 | 0.80 (0.54-1.20)  .286 |
| Housewife/  househusband | 0.53 (0.21-1.39)  .198 | 0.83 (0.35-1.99)  .684 | 0.78 (0.33-1.85)  .580 |  | 0.97 (0.63-1.48)  .884 | 0.82 (0.62-1.10)  .184 | 0.87 (0.57-1.33)  .530 |
| Manual workers | 0.56 (0.23-1.34)  .190 | 0.85 (0.40-1.84)  .683 | 0.61 (0.28-1.33)  .213 |  | - 1. (0.68-1.52)   .949 | 0.85 (0.65-1.12)  .259 | 1.14 (0.77-1.68)  .511 |
| Others | 0.86 (0.35-2.09)  .740 | 0.76 (0.34-1.70)  .512 | 0.41 (0.17-0.97)  **.043** |  | 0.95 (0.62-1.45)  .811 | 1.10 (0.83-1.45)  .521 | 1.05 (0.70-1.57)  .817 |
| Unemployed | 1 | 1 | 1 |  | 1 | 1 | 1 |

Table S11. Proportions of STI-related microorganisms in male cases and controls

| **Table S11.** **Proportions of STI-related microorganisms in male cases and controls** | | | | | | | | | | |  |
| --- | --- | --- | --- | --- | --- | --- | --- | --- | --- | --- | --- |
| **Micro-organism** | **Controls**  **N=1176** | **Urethral serous discharge**  **N=183** | **Urethral purulent discharge**  **N=161** | **Scrotum swelling or pain**  **N=68** | **Epididymis swelling or pain**  **N=35** | **Balanopos-thitis**  **N=74** | **Urethral burning or irritation**  **N=619** | **Genital or perianal blisters**  **N=72** | **Genital or perianal ulcers**  **N=52** | **Genital or perianal warts**  **N=182** | |
| *N. gonorrhoeae*  Negative  Positive | 1109 (94.3)  67 (5.7) | 151 (82.5)  32 (17.5) | 102 (63.4)  59 (36.6) | 63 (92.6)  5 (7.4) | 33 (94.3)  2 (5.7) | 67 (90.5)  7 (9.5) | 536 (86.6)  83 (13.4) | 63 (87.5)  9 (12.5) | 44 (84.6)  8 (15.4) | 168 (92.3)  14 (7.7) | |
| *C. trachomatis*  Negative  Positive | 1092 (92.9)  84 (7.1) | 139 (76.0)  44 (24.0) | 122 (75.8)  39 (24.2) | 63 (92.6)  5 (7.4) | 32 (91.4)  3 (8.6) | 66 (89.2)  8 (10.8) | 505 (81.6)  114 (18.4) | 63 (87.5)  9 (12.5) | 47 (90.4)  5 (9.6) | 165 (90.7)  17 (9.3) | |
| *T. vaginalis*  Negative  Positive | 1170 (99.5)  6 (0.5) | 181 (98.9)  2 (1.1) | 159 (98.8)  2 (1.2) | 68 (100.0)  0 (0) | 34 (97.1)  1 (2.9) | 73 (98.6)  1 (1.4) | 617 (99.7)  2 (0.3) | 72 (100.0)  0 (0) | 52 (100.0)  0 (0) | 182 (100.0)  0 (0) | |
| *M. genitalium*  Negative  Positive | 1152 (98.0)  24 (2.0) | 171 (93.4)  12 (6.6) | 152 (94.4)  9 (5.6) | 67 (98.5)  1 (1.5) | 35 (100.0)  0 (0) | 73 (98.6)  1 (1.4) | 592 (95.6)  27 (4.4) | 70 (97.2)  2 (2.8) | 52 (100.0)  0 (0) | 177 (97.3)  5 (2.7) | |
| HSV-1  Negative  Positive | 1160 (98.6)  16 (1.4) | 176 (96.2)  7 (3.8) | 157 (97.5)  4 (2.5) | 67 (98.5)  1 (1.5) | 35 (100.0)  0 (0) | 73 (98.6)  1 (1.4) | 606 (97.9)  13 (2.1) | 70 (97.2)  2 (2.8) | 51 (98.1)  1 (1.9) | 179 (98.4)  3 (1.6) | |
| HSV-2  Negative  Positive | 1167 (99.2)  9 (0.8) | 178 (97.3)  5 (2.7) | 161 (100.0)  0 (0) | 68 (100.0)  0 (0) | 35 (100.0)  0 (0) | 74 (100.0)  0 (0) | 610 (98.5)  9 (1.5) | 67 (93.1)  5 (6.9) | 49 (94.2)  3 (5.8) | 179 (98.4)  3 (1.6) | |
| *M. hominis*  Negative  Positive | 1109 (94.3)  67 (5.7) | 170 (92.9)  13 (7.1) | 151 (93.8)  10 (6.2) | 65 (95.6)  3 (4.4) | 29 (82.9)  6 (17.1) | 67 (90.5)  7 (9.5) | 571 (92.2)  48 (7.8) | 63 (87.5)  9 (12.5) | 49 (94.2)  3 (5.8) | 170 (93.4)  12 (6.6) | |
| *U. parvum*  Negative  Positive | 952 (81.0)  224 (19.0) | 157 (85.8)  26 (14.2) | 145 (90.1)  16 (9.9) | 50 (73.5)  18 (26.5) | 29 (82.9)  6 (17.1) | 64 (86.5)  10 (13.5) | 516 (83.4)  103 (16.6) | 62 (86.1)  10 (13.9) | 43 (82.7)  9 (17.3) | 148 (81.3)  34 (18.7) | |
| *U. urealyticum*  Negative  Positive | 1050 (89.3)  126 (10.7) | 153 (83.6)  30 (16.4) | 141 (87.6)  20 (12.4) | 61 (89.7)  7 (10.3) | 30 (85.7)  5 (14.3) | 61 (82.4)  13 (17.6) | 548 (88.5)  71 (11.5) | 51 (70.8)  21 (29.2) | 45 (86.5)  7 (13.5) | 160 (87.9)  22 (12.1) | |

Table S12. Proportions of STI-related microorganisms in female cases and controls

| **Table S12.** **Proportions of STI-related microorganisms in female cases and controls** | | | | | | | | | |
| --- | --- | --- | --- | --- | --- | --- | --- | --- | --- |
| **Micro-organism** | **Controls**  **N=1966** | **Abnormal vaginal discharge**  **N=2917** | **Mucopurulent cervicitis**  **N=260** | **Lower abdominal pain**  **N=492** | **Vaginal itch**  **N=79** | **Urethral burning or irritation**  **N=251** | **Genital or perianal blisters**  **N=41** | **Genital or perianal ulcers**  **N=34** | **Genital or perianal warts**  **N=100** |
| *N. gonorrhoeae*  Negative  Positive | 1934 (98.4)  32 (1.6) | 2858 (98.0)  59 (2.0) | 255 (98.1)  5 (1.9) | 482 (98.0)  10 (2.0) | 78 (98.7)  1 (1.3) | 246 (98.0)  5 (2.0) | 39 (95.1)  2 (4.9) | 32 (94.1)  2 (5.9) | 97 (97.0)  3 (3.0) |
| *C. trachomatis*  Negative  Positive | 1780 (90.5)  186 (9.5) | 2614 (89.6)  303 (10.4) | 229 (88.1)  31 (11.9) | 443 (90.0)  49 (10.0) | 70 (88.6)  9 (11.4) | 225 (89.6)  26 (10.4) | 36 (87.8)  5 (12.2) | 29 (85.3)  5 (14.7) | 90 (90.0)  10 (10.0) |
| *T. vaginalis*  Negative  Positive | 1934 (98.4)  32 (1.6) | 2851 (97.7)  66 (2.3) | 248 (95.4)  12 (4.6) | 486 (98.8)  6 (1.2) | 78 (98.7)  1 (1.3) | 242 (96.4)  9 (3.6) | 39 (95.1)  2 (4.9) | 32 (94.1)  2 (5.9) | 99 (99.0)  1 (1.0) |
| *M. genitalium*  Negative  Positive | 1926 (98.0)  40 (2.0) | 2850 (97.7)  67 (2.3) | 253 (97.3)  7 (2.7) | 479 (97.4)  13 (2.6) | 75 (94.9)  4 (5.1) | 243 (96.8)  8 (3.2) | 40 (97.6)  1 (2.4) | 34 (100.0)  0 (0) | 94 (94.0)  6 (6.0) |
| HSV-1  Negative  Positive | 1953 (99.3)  13 (0.7) | 2859 (98.0)  58 (2.0) | 254 (97.7)  6 (2.3) | 480 (97.6)  12 (2.4) | 78 (98.7)  1 (1.3) | 238 (94.8)  13 (5.2) | 38 (92.7)  3 (7.3) | 33 (97.1)  1 (2.9) | 99 (99.0)  1 (1.0) |
| HSV-2  Negative  Positive | 1950 (99.2)  16 (0.8) | 2864 (98.2)  53 (1.8) | 256 (98.5)  4 (1.5) | 485 (98.6)  7 (1.4) | 79 (100.0)  0 (0) | 240 (95.6)  11 (4.4) | 39 (95.1)  2 (4.9) | 32 (94.1)  2 (5.9) | 98 (98.0)  2 (2.0) |
| *M. hominis*  Negative  Positive | 1732 (88.1)  234 (11.9) | 2464 (84.5)  453 (15.5) | 214 (82.3)  46 (17.7) | 429 (87.2)  63 (12.8) | 60 (75.9)  19 (24.1) | 220 (87.6)  31 (12.4) | 31 (75.6)  10 (24.4) | 27 (79.4)  7 (20.6) | 80 (80.0)  20 (20.0) |
| *U. parvum*  Negative  Positive | 969 (49.3)  997 (50.7) | 1417 (48.6)  1500 (51.4) | 139 (53.5)  121 (46.5) | 251 (51.0)  241 (49.0) | 35 (44.3)  44 (55.7) | 148 (59.0)  103 (41.0) | 20 (48.8)  21 (51.2) | 13 (38.2)  21 (61.8) | 51 (51.0)  49 (49.0) |
| *U. urealyticum*  Negative  Positive | 1706 (86.8)  260 (13.2) | 2477 (84.9)  440 (15.1) | 209 (80.4)  51 (19.6) | 421 (85.6)  71 (14.4) | 65 (82.3)  14 (17.7) | 202 (80.5)  49 (19.5) | 35 (85.4)  6 (14.6) | 26 (76.5)  8 (23.5) | 80 (80.0)  20 (20.0) |
